# Predictors of adherence to isoniazid preventive therapy among HIV positive adults in Addis Ababa, Ethiopia

**DOI:** 10.1186/1471-2458-11-916

**Published:** 2011-12-12

**Authors:** Mesele Mindachew, Amare Deribew, Fasil Tessema, Sibhatu Biadgilign

**Affiliations:** 1 Department of General Public Health, College of Public Health and Medical Science, Jimma University, Jimma, Ethiopia; 2 Department of Epidemiology, College of Public Health and Medical Science, Jimma University, Jimma, Ethiopia

**Keywords:** Adherence, TB, Isoniazid Preventive Therapy, HIV, Ethiopia

## Abstract

**Background:**

Isoniazide preventive therapy (IPT) is given to individuals with latent infection of tuberculosis (TB) to prevent the progression to active disease. One of the primary reasons for failure of IPT is poor adherence.

**Methods:**

A cross sectional study was conducted in four hospitals in Addis Ababa. Data were collected using a pre-tested interviewer-administered structured questionnaire. Bivariate and multivariate analysis was done to identify predictors of IPT.

**Results:**

A total of 319 (97.5%) individual participated in this study. Within seven days recall period, self-reported dose adherence rate was 86.5%. Individual who received explanation about IPT from health care providers (OR = 7.74; 95%CI: 3.144, 19.058); who had good feeling/comfortable to take IPT in front of other people [OR = 5.981, 95%CI (2.308, 15.502)] and who attended clinical appointment regularly (OR = 4.0; 95%CI: 1.062, 15.073) were more likely to adhere to IPT. Participants who developed IPT related adverse effect were 93% less likely to adhere to the prescribed doses (OR = 0.065; 95%CI: 0.024, 0.179).

**Conclusion:**

The prevalence of self reported dose adherence over the past 7 days was higher. Non-adherence was observed among respondent who were not provided with sufficient information about IPT. The health care providers need to strengthen their educational and counseling efforts to convince the patient before putting them on IPT. To enhance adherence, health education efforts should focus on the importance of IPT, the details of the regimen and adverse effects.

## Background

Tuberculosis is among the top ten causes of global mortality and morbidity. Recently, 32% of the world's population is infected with TB. Over 95% of new tuberculosis cases and deaths occur in developing countries where the highest incidence and number of deaths occur in Asia and sub-Saharan Africa [1]. HIV/AIDS is the greatest risk factors for the development of TB. The risk of reactivation of latent infection of TB is greatly increased in Africa as a result of the HIV/AIDS epidemic [2-5].

The World Health Organization (WHO) had proposed a framework of TB/HIV/AIDS collaborative activities to prevent the occurrence of TB-HIV disease. IPT, intensified TB case finding, and infection prevention are the major strategies to control TB in HIV-positive individuals [6]. Cognizant of this fact, the Ethiopia national TB/HIV collaboration program adopted these activities including IPT. The Ethiopian national TB guideline recommends that Isoniazide (INH) should be given to patients daily at a dose of 5 mg/kg, a maximum dose of 300 mg/day for a period of 6 months. Pyridoxine at a fixed daily dose of 25 mg is indicated in order to reduce the risk of developing INH-induced peripheral neuropathy [7]. Tuberculosis preventive therapy should only be considered in areas that have a good functioning of tuberculosis program [8]. There is now strong evidence from several randomized controlled trials about the efficacy of preventive therapy in the prevention of tuberculosis in persons infected with the human immunodeficiency virus. However, one of the primary reasons for failure of IPT is poor adherence. Accurate assessment of adherence and its associated factor is a necessary first step towards improving adherence to IPT [9]. There is limited knowledge in Ethiopia about the rate of adherence to IPT and its determinants. The aim of this study was to address this knowledge gap using a multi-center study in Addis Ababa, Ethiopia.

## Methods

### Study setting and participants

A cross sectional study was conducted in Yekatit 12, Zewditu, Gandi and Minilik hospitals in Addis Ababa from February 1 to March 30, 2010. The study hospitals are among the biggest in Ethiopia and serving the majority of the HIV/AIDS clients in the country. The hospital records showed that from the total of 1808 adults taking IPT in the whole Ethiopia, 1303 of them were attending in these hospitals.

The source populations were People Living with HIV/AIDS (PLHA) without active TB attending the ART clinics of the study hospitals and, who were on IPT and on follow up during the study period. The study population was sampled adults who were receiving IPT and fulfilling the inclusion criteria in the study hospitals. Adult (> = 18 years) HIV positive individuals who were taking IPT for at least one month before the initiation of the study were included in the study. Individuals who had been taking IPT for less than a month and/or were seriously sick were excluded from the study.

### Sample size and sampling technique

The sample size was calculated using Epi-Info 6.04 statistical software with the assumptions of adherence rate of 50%, 5% margin of error and 95% confidence interval and 10% for non-response rate. This gave us a total sample size of 327. This sample size was distributed to the selected hospitals according to the number of patients proportionally. All HIV positive individuals attending the TB/HIV clinics of the selected hospitals and who fulfilled the entry criteria were identified from clinics using their identification number as a sampling frame. From the sampling frame, participants were selected using simple random sampling technique.

### Data collection process

Data were collected by trained public health officers using a structured and pre-tested questionnaire. The questionnaire contained dependent and independent variables and was originally developed in English; and then translated into Amharic and back translated to English by other person who was blind to the original questionnaire to check for its consistency. The dependent variable was self-reported adherence to IPT in the last 3, 7 and 30 days. The independent variables included: socio-demographic variables, medication adverse effect, emotional and social support, convenience of regimen (the duration, dose, and frequency), knowledge about IPT and understanding the important of adherence; belief on the efficacy of medication; substance use such as *Khat*(Catha Edulis), alcohol, and cigarette; patient and physician relationship.

The percent of adherence was calculated for each drug by dividing the number of pills taken by the number of pills prescribed. Then, the percent of adherence to the IPT was estimated by the average adherence rate to the drugs.

### Data analysis

The data were entered into SPSS 16.0, edited, and cleaned thoroughly and analyzed. The analysis consisted of basic summaries of patient characteristics, bivariate and multivariate analysis of the relation between adherence and various factors. All explanatory variables that were significantly associated with the outcome variable in the bivariate analyses (*P *< 0.05) were entered in stepwise logistic regression model to identify independent predictor of adherence. In this study, patients who report an intake of 80% or more of the prescribed medication over past 3 and 7 days were considered to be adherent, while a patient who reported an intake of less than 80% of the prescribed doses over the past 3 and 7 days were considered to be non adherent [10]. Adverse effect was also defined as an unwanted effect caused by the administration of INH such as numbness as a result of neuropathy, sign of liver toxicity, rash, nausea, and seizure. Ethical Clearance was obtained from Institutional Ethical Review Committee of Jimma University and ethical review committee of Addis Ababa Health Bureau.

## Results

### Characteristics of the study subjects

Of the proposed study participants, 319 (97.5%) were included in this study. One hundred and twenty-four (38.9%) were males and their age ranged from 22 to 71 years with the mean (SD) age of 38(± 10.6). Orthodox was the dominant religion (84.3%) and 125 (39.2%) were married. Almost half 172(53.9%) of the respondents were Amhara, and more than half of the respondent 203(63%) had average monthly income of less than 35.5 USD (1US $ = 17Birr), 94(29.5%) were grade 1-8 educational level, and 114(35.7%) were private institution employee (Table 1).

**Table 1:** Basic socio-demographic attributes of the cross-sectional survey at Addis Ababa governmental hospitals, Addis Ababa, May 2010

Socio-demographic Variables	Frequency (%)
**Sex (n = 319)**	

**Male**	124(38.9%)

**Female**	195 (61.1%)

**Age 38.7 (+ 10.6) yrs (n = 319)**	

**< 25**	16(5)

**25-34**	110 (34.5)

**35-44**	85 (26.6)

**≥45**	108 (33.9)

**Ethnicity (n = 319)**	

**Oromo**	71 (22.3)

**Ahmara**	172 (53.9)

**Tigre**	48(15)

**Others^#^**	28 (8.8)

**Religion (n = 319)**	

**Orthodox**	269 (84.3)

**Protestant**	33 (10.3)

**Muslim**	15(4.7)

**Catholic**	2(0.6)

**Marital status (n = 319)**	

**Single**	66(20.7)

**Married**	125 (39.2)

**Divorced**	39 (12.2)

**Widowed**	89 (27.9)

**Educational status(n = 319)**	

**Illiterate**	40 (12.5)

**Grade 1-8**	94 (29.5)

**Grade 9-10**	39 (12.2)

**Grade +10 and certificate**	93 (29)

**Diploma and above**	52 (16.3)

**Occupational status(n = 319)**	

**House wife**	60 (18.8)

**Student**	3 (0.9)

**Government employment**	44 (13.8)

**Private employee**	114 (35.7)

**Merchant**	14(4.4)

**Driver**	5 (1, 6)

**No jobs**	71(22.3)

Others*	8(2.3)

**Monthly income(n = 319) in USD^$^**	

**> = 35.5**	203(63.6)

**< 35.5**	116(36.4)

#Affar, Guragie, Somali, Welaita

*Clerical, Sales and services, Manual labor, Agriculture

^$^Exchange rate 1 USD = 17 Ethiopian Birr (ETB)

### Patient report on adherence to IPT

**Figure 1:**
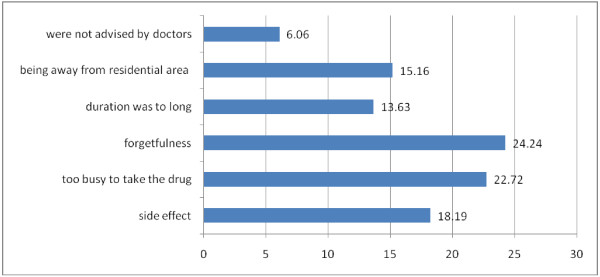
**Reasons given for missing to take IPT among people living with HIV in Addis Ababa government hospitals, Addis Ababa, May 2010**.

As shown in Table 2, the 3 days, 7 days, and the past 1 month self-reported adherence rate was 281(88.1%), 276 (86.5%), and 255 (79.9%) respectively. The average [SD] number of doses missed in the past 3 days, 7 days and 1 month prior to the study was 1.84[SD ± 0.75], 2.64[SD ± 0.1.71] and 6.3[SD ± 7.31] respectively. Of the total participants, 66 (20.7%) had history of missed days (missed one or more doses of isoniazide in the past 3 days, 7 days, in the last 1 month prior to enrolment in study)(Table 3). The most frequently mentioned reasons for missing pills were side effect (18.19%, N = 12), being too busy to take the drug (22.72%, N = 16), forgetfulness (24.24%, N = 15), perceived long duration of treatment (13.63%, N = 9), being away from residential area (15.16%, N = 10) and lack of advice by doctors (6.06%, N = 4) (Figure 1).

**Table 2:** IPT adherence status by self-reports of the cross-sectional survey at Addis Ababa governmental hospitals, Addis Ababa, May 2010

Duration	Adherent Frequency (%)	Non-adherent Frequency (%)	Total Frequency (%)
Past three days	281(88.1)	38(11.9)	319(100)

Past 7 days	276(86.5)	43(13.5)	319(100)

Past one month	255(79.9)	64(20.1)	319(100)

**Table 3:** Frequency table that shows patient's adherence practice of the cross-sectional survey at Addis Ababa governmental hospitals, Addis Ababa, May 2010

Variables	Frequency (%)
**History of missed days**	

**Yes**	66(20.7)

**No**	253(79.3)

**Time of missing prior to interview to interview**	

Within the first week	29(51.8)

Between 1-2 month	17(30.4)

Between 3-5 months	4(7.1)

Above 5 months	2(3.6)

I don't know	4(7.1)

### Factors independently associated with adherence to IPT

Individual who had receive explanation about IPT were 8 times more likely to be adherent as compared to those who did not receive (OR = 7.740; 95%) CI: (3.144, 19.058). Patients who developed IPT related adverse effects were 93% less likely to adhere to the prescribed doses (OR = 0.065; 95%CI: 0.024, 0.179). Study participants who had good feeling/comfortable to take IPT drug in front of others people were 6 times more likely to adhere to the prescribed medication (OR = 5.981; 95%CI: 2.308, 15.502) and those who attend clinical appointment regularly were 4 times more likely to adhere to IPT compared to those who did not attend regularly (OR = 4.001; 95%CI: 1.062, 15.073) (Table 4).

**Table 4:** Independent predictors of adherence to IPT among people living with HIV in Addis Ababa government hospitals, Addis Ababa, May 2010

Variable	Adherent Frequency (%)	Non-adherent Frequency (%)	Crude/unadjusted OR(95%CI)	p-value	Adjusted OR(95%CI)	p-value
**Did the regimen explained to you**						
**Yes**	227(93)	17(7)	**7.085(3.57, 14.05)**	**< 0.001**	**7.74(3.14, 19.06)**	**< 0.001**
**No**	54(72)	21(28)	**1**		**1**	
**Adverse effect**						
**Yes**	19(55.9)	15(44.1)	**0.07(0.03, 0.16)**	**< 0.001**	**0.06(0.02, 0.18)**	**< 0.001**
**No**	262(91.9)	23(8.1)	**1**		**1**	
**Feel comfortable to take drug in front of others**						
**Yes**	169(98.8)	28(1.2)	**7.53(3.24, 17.52)**	**< 0.001**	**5.98(2.31, 15.50)**	**< 0.001**
**No**	112(75.7)	10(24.3)	**1**		**1**	
**Attend clinical appointment regularly**						
**Yes**	265(90.1)	29(9.9)	**4.30(1.76, 10.49)**	**< 0.001**	**4.00(1.06, 15.07)**	**0.04**
**No**	15(62.5)	9(37.5)	**1**		**1**	

## Discussion

We tried to assess the rate of adherence to IPT and its determinants among HIV positive adults in the capital city of Ethiopia. We use self-reported dose adherence due to its ease of practicality, low cost, readiness to obtain the desired information, and to identify patients' at risk for non-adherence [11]. This study revealed an overall prevalence rate of IPT adherence rate of 276[86.5%] over the past 7 days prior to the interview. In the operational assessment of adherence among HIV infected individual in Uganda, adherence rate of 38% was reported [11]. A facility based cross-sectional study in rural South Africa also found that only 47.1% were adherent to isoniazid preventive therapy [12]. In a prospective cohort study in Thailand using daily isoniazid for 6 months, 31% failed to complete the 9-month regimen [13]. In contrast to these, a cross-sectional study in South Africa revealed relatively similar finding with the present study with overall rate of self reported adherence rate of 72%[14] and a randomized control trail of adherence to IPT among HIV patient in Uganda also showed similar finding with adherence rate of 88% [15]. This may be due to the fact that majority of the respondent had receive the information about isoniazide preventive therapy and perceived benefit of the medication. In addition, it might be also due to the great emphasis given to the TB-HIV collaborative activity by the government and implementing partners. In the current study, different reasons for missing pills such as forgetfulness, being buss, and away from home were reported which is consistent with the findings of other studies [16,17]. In our study, patients' who didn't miss clinic appointment were more likely to complete their regimen which is similar to a study elsewhere [18].

According to a study conducted in Kenya and Zambia, the poor quality of physicians' interpersonal skills has been shown to negatively affect adherence [19,20]. Four cross-sectional studies showed the impact of poor patient-physician relation on adherence in different setting [21-24]. Randomized trial also showed that an increase in non-adherence in situations where doctors appear insensitive, use medical jargon, view patients as complainers, or do not provide clear messages about the cause of the illness or reasons for treatment [25,26]. Stigma or uncomfortable to take drug in front of others was one of the factors for adherence to IPT. A stigma attached to TB in China leads to imposition of socio-physical distance and participatory restrictions on those suffering from the disease [27] and social and family support positively influenced adherence [12] and reasons for low adherence to preventive INH treatment, other than side effects, include fear of stigma [18,28]. Patients' who developed side effect were less likely to complete the prescribed regimen. A systematic review of randomized trial showed that adherence has been shown to decrease with increased concerns about the toxicity of anti-TB medications and fear of side effects [15]. It is possible that minor adverse effects, which did not pose a danger in the clinician's view, nevertheless resulted in discomfort and contributed to a patients' decision not to continue treatment [29] and their completion rate was worse among those with side effects [30,31]. Patient reports of minor treatment side effects should alert clinicians to reassess and discuss the benefits and the risks of therapy as patient beliefs about the necessity of therapy and concerns about potential adverse effects is a stronger predictor of medication adherence than clinical or socioeconomic factors [32]. Knowledge about the regimen and the disease has significant association with adherence which was inconsistent with the study conducted in Uganda [15]. A cross-sectional study in South Africa also showed no significant association between patients' knowledge about the treatment and the disease with adherence [14]. Knowledge and attitudes towards tuberculosis were not significant predictors in United States [33]. A study conducted to assess factors contributing for poor compliance also showed that knowledge and attitude towards tuberculosis were not significant predictors of adherence [34]. However, other studies reported that beliefs and attitudes may be more important than side effects in predicting adherence and influencing health behavior [18,35,36]. Studies reported that patient's compliance with therapy is affected by issues of belief, health motivation, perceived susceptibility to disease and its severity, views in the benefit of professional intervention and knowledge of the condition [37,38]. It has been documented that when patients' know about the importance of complying with drug therapy and their adherence to the prescribed regimen is improved [39]. In similar study in India, it was found that there was an association between the compliance behavior of the patients' and their knowledge of specific aspects of the disease [40]. patients' who perceive their illness to be more serious and who believe that treatment will alleviate the condition are more likely to be compliant [41]. These discrepancies/inconsistencies among different studies may be due to difference on educational status, health service coverage, and access to health related information and level of economic growth across countries. Poor knowledge about treatment regimens and patient perception of benefits obtained from therapy was significantly associated with non adherence [12,40,42-45]. This might be due to differences in the study design. Knowledge of factors associated with poor compliance can help to identify groups at risk of defaulting and lead to improved patients' education. Belief in isoniazide (INH) safety was associated with a positive test and patients who believed INH is important for prevention of TB disease were more likely to be negative urine results [14]. Completers were motivated by understanding the importance of IPT and counseling [28].

This study had some limitations. First, since there is no a "gold standard" for assessment of adherence, we used self-reported adherence which might introduce recall bias. Second, the cross sectional nature of our study may not establish cause effect relationship. Despite the above limitations, the study had several strengths, including using a relatively large sample size, inclusion of several sites, inclusion of several variables and review of available medical charts.

## Conclusion

Adherence rate in Addis Ababa is higher than most studies in Africa. However, stigma, lack of provider-client communication, irregular clinic attendance and fear of side effects of the medication were the best predictor's of non-adherence. Health workers and the ministry of health should design and deliver tailored health education messages on the aforementioned risk factors for clients to promote better adherence.

## Competing interests

The authors declare that they have no competing interests.

## Authors' contributions

MM conceived and designed the study and collected data in the field, performed analysis, interpretation of data, and draft the manuscript. AD assisted with the design, interpretation of data and the critical review of the manuscript. FT participated in design and helped to draft the manuscript and critically reviewed the manuscript. SB assisted with the design, interpretation of data, draft and critical review of the manuscript. All authors approved and read the final manuscript. All authors participated in critical appraisal and revision of the manuscript.

## Pre-publication history

The pre-publication history for this paper can be accessed here:

http://www.biomedcentral.com/1471-2458/11/916/prepub
